# A realist evaluation to identify targets to improve the organization of compression therapy for deep venous thrombosis- and chronic venous disease patients

**DOI:** 10.1371/journal.pone.0272566

**Published:** 2022-08-08

**Authors:** Rachel H. P. Schreurs, Manuela A. Joore, Daisy P. De Bruijn-Geraets, Hugo ten Cate, Arina J. ten Cate-Hoek

**Affiliations:** 1 Thrombosis Expert Centre Maastricht and Laboratory for Clinical Thrombosis and Haemostasis, Maastricht, The Netherlands; 2 Cardiovascular Research Institute Maastricht (CARIM), Maastricht, The Netherlands; 3 Department of Internal Medicine, Maastricht University Medical Centre, Maastricht, The Netherlands; 4 Department of Clinical Epidemiology and Medical Technology Assessment, Maastricht University Medical Center, Maastricht, The Netherlands; 5 CAPHRI Care and Public Health Research Institute, Maastricht University, Maastricht, The Netherlands; Erasmus Universiteit Rotterdam, NETHERLANDS

## Abstract

**Background:**

Although compression therapy is well established for patients with deep venous thrombosis (DVT) and chronic venous disease (CVD), considerable variation exists in its organization in clinical practice which may impact patient outcomes. The current study aims to deepen our understanding of the main drivers of the complex care organization for compression therapy and to identify targets for improvement.

**Methods:**

This realist evaluation includes a mixed-method design consisting of semi-structured interviews with patients and health care professionals involved in compression therapy (n = 30), stakeholder meetings (n = 2) and surveys (n = 114). Data were collected to create the content of context-mechanism-outcome-configurations (CMOcs) important in compression therapy. Based on these CMOcs, targets for improvement to optimize the organization of compression care were identified.

**Results:**

We identified overarching context factors and mechanisms targeting four optimal outcomes for the organization of compression therapy: selecting initial compression therapy types that support patient’s self-reliance (1), evidence based selection of elastic compression stocking type and class (2), patient-based selection of assistive devices (3), individualizing treatment duration for DVT patients (4a) and providing follow-up for CVD patients (4b). We found that increasing health care professionals’ knowledge of compression therapy, the availability of unambiguous protocols and guidelines, increasing patient involvement (and if applicable their informal care giver) in the decision making process, the accessible availability of resources, and increasing interdisciplinary consultation enhanced desirable outcomes. These targets triggered mechanisms such as increased health care professionals’ willingness, confidence and motivation to provide patient-based care and increased patients’ self-confidence and self-efficacy.

**Conclusions:**

This study provides a detailed insight into what needs to be in place to optimize compression care and identified five main targets for improvement.

## Introduction

Compression therapy is an effective therapy used to treat acute symptomatology and prevent complications such as post-thrombotic syndrome for deep venous thrombosis (DVT) [1–3] and ulcer development for chronic venous disease (CVD) [4, 5]. Both diseases are frequently encountered worldwide; 1–2 per 1000 persons annually develop a DVT [6] and the prevalence of CVD in the population ranges from 13.7% to 19.7% [7–9].

The organization of compression therapy is complex and involves collaboration between the patient and various healthcare professionals. There is considerable variation in how compression therapy is organized for both DVT and CVD patients [10]. In this study, this variability was found to affect patient’s chances to remain self-reliant, the advised treatment duration for DVT patients, and was expected to affect compliance with compression therapy. Compliance with compression therapy is a major determinant of effectiveness [3, 11]. Not being able to independently apply or remove the elastic compression stockings (ECS) was found to be an important reason for non-compliance [12]. On the other hand, it was found that in some regions a subset of all patients is treated longer than necessary without added benefits [2, 10]. Decreasing variability in the organization of compression therapy is necessary to improve therapy and is likely to result in an increase in desirable outcomes.

With this study we used a realist evaluation to deepen our understanding of the main drivers in the complex organization of compression therapy for patients with DVT and CVD and identify targets for improvement. Realist evaluation acknowledges and identifies the complexity of a system by identifying how outcomes are achieved [13, 14]. It is shown that a realist evaluation is a well suited approach to investigate complex causal pathways, and can advance understanding of the best circumstances for increasing improvement strategies’ impact [15, 16].

## Methods

### General study design

We performed a realist evaluation using a mixed-methods design. In this evaluation, we explore how different patterns result in specific outcomes by asking the explanatory question: what works (or does not work), for whom, and under which circumstances. This was achieved by the identification of causal explanations in the complex dynamics among context and mechanisms that interact and contribute to context-mechanism-outcome configurations (CMOcs) [13, 16–19]. We used the definitions for context, mechanism, and outcome as introduced by Pawson and Tilley [14]. Context includes all individual capacities of the patients and professionals, their interpersonal relationships, institutional settings, and infrastructural or welfare systems affecting daily practice. Mechanisms generate outcomes and include causal forces as well as choices, reasoning, and decisions made in response to the context and resources provided. Outcomes are the effects produced by causal mechanisms triggered in a specific context. We reported this evaluation using the RAMESES publication standards [20].

We only included CVD patients that were conservatively treated and excluded CVD patients with an active venous ulcer since these patients have different care- and treatment needs compared to the patients selected for the current study. Our study was assessed by the Ethics Board of Maastricht University Medical Center (MUMC), Maastricht (2019–1125), and was considered not to be subject to the Medical Research involving Human Subjects Act (WMO).

### Data collection: Interviews, stakeholder meetings, and surveys

The study context, selection of interviewees and stakeholders were described in the FRAM (Functional Resonance Analysis Method) study we previously conducted [10]. We performed the study in two regions in the Netherlands: Limburg and North-Holland. We purposively selected these regions based on their geographical spread and the differences in hospital setting (an academic hospital in Limburg and a general hospital with two locations in North-Holland). Furthermore, several interventions to improve self-reliance had already been implemented in North-Holland, while a tailored ECS treatment duration was implemented by internists in Limburg. This allowed us to evaluate factors contributing to the differences in implementation of these specific interventions in the two regions.

RS conducted thirty semi-structured interviews (n = 15 for each region) with purposively recruited experienced professionals (n = 25) and patients (n = 5) from the two selected regions. Professionals were recruited beginning with a senior policy adviser from a home care organization in Limburg, and a senior project manager from a home care organization in North-Holland. Consecutive interviewees recruited other experienced professionals and patients until during three consecutive interviews no new context factors or mechanisms emerged indicating that data was saturated [21]. Patients were selected by health care professionals and included self-reliant patients and patients who required home care assistance for both DVT and CVD. Interviews with professionals were conducted by telephone whereas patients were interviewed either by telephone or at the patient’s home. All interviewees were informed regarding the goal of the interview and consented verbally to participate. We audio-recorded and transcribed the interviews verbatim.

A predesigned interview template was used to guide the interviews as presented in S1 File. In brief, interviewees were asked to define important process outcomes that enhance treatment effect and patients’ self-reliance. To better understand how context and generative mechanisms affected these outcomes, interviewees were asked how they explained these outcomes, and what factors affected their actions in different contexts. Following the interviews, we selected key stakeholders among the interviewees (one per discipline) based on clinical experience. Two stakeholder meetings were organized (n = 8 participants for Limburg and n = 9 for North-Holland) to refine and validate the CMOcs as identified in the interviews and discuss potential new insights.

To quantify the main process outcomes, we used Qualtrics (Qualtrics, Provo, UT, version September 2020) to administer online surveys among a larger sample of professionals. For these surveys, we purposively sampled the professional groups based on the extent and influence of their activities within the entire process, and the degree of variability in the performance of the activity described. These professionals included occupational therapists, medical stocking suppliers, home care nurses, and general practitioners (GPs). Although internists and dermatologists also contributed significantly to the process, we decided to not include them in the survey, since only a small proportion of internists and dermatologists perform DVT- or CVD care in the selected hospitals (these professionals were included in the semi-structured interviews and stakeholder meetings).

In total 14 practices for occupational therapy, 9 medical stocking companies, the main home care organization of each region, and 33 GP-practices were invited to participate in the online surveys. As there is no registration or centrally held record of professionals working in compression care, clinical practices were found by searching Google Maps and subsequently contacted by email or telephone. They were asked whether they were willing to participate, to give an indication of the number of professionals providing compression care in their organization, and to distribute the survey among these professionals. Two reminders were sent after the initial invitation.

The first author (RS) developed the questions and response options in the survey in discussion with co-authors (ATC, MJ) and aimed to quantify the process outcomes. The surveys consisted of 8–19 questions depending on the professional group (as presented in S2 File). Pilot testing was undertaken by sending the survey to one stakeholder per professional group, feedback was incorporated in the final survey designs.

### Data-analysis

The semi-structured interviews, the stakeholder meetings, and the responses to the open-ended survey questions were coded and categorized as context (C), mechanism (M), or outcome (O) using a codebook based on the definitions as described by Pawson and Tilley [14]. Outcomes were selected if stakeholders considered them to be important for further exploration to optimize the organization of care and if they were expected to improve treatment effect and/or patient´s self-reliance.

RS summarized and coded all interviews, stakeholder meetings, and open-ended survey questions after which the results were repeatedly discussed with MJ, ATC, and DDB. If information was missing or not clear, stakeholders were contacted to provide further information. Common links and consistent patterns between context, mechanisms, and outcomes across the data were identified to generate the CMOcs. All CMOcs targeted one of the four phases of compression therapy being: initial compression therapy (in which the patient uses either multilayer compression bandages, temporary compression hosieries or adjustable compression bandages until edema has receded), onset of ECS therapy (in which the ECS is fitted and delivered), implementation of assistive devices (in which the patient is trained how to use assistive devices upon indication), and long-term maintenance of ECS therapy (in which follow-up takes place and ECS duration is determined) [10].

When patient volumes showed large variation within a region, local stakeholders were asked to indicate whether patient volumes were representative in their opinion and how these results could be explained.

To identify what works or does not work, for whom, and under which circumstances, we identified overarching contextual factors and mechanisms that occurred multiple times in our CMOcs and resulted in desirable outcomes. Next, we identified the CMOcs that covered the same subject and resulted in undesirable outcomes. We considered the context factors and mechanisms that differed between those CMOcs and the context factors and mechanisms resulting in desirable outcomes as targets for improvement. Moreover, we examined all other CMOcs to check whether additional targets could be identified.

We used descriptive statistics to analyze the quantitative survey results for rating scale questions in Microsoft Excel 2010.

## Results

In addition to the 30 semi-structured interviews and two stakeholder meetings (with a total of 17 participants), 114 surveys were received (overall response rate 74%). An overview of the interviewees per discipline and response rates to the survey are further specified in S1 Table.

Seven CMOcs emerged from our data. A detailed description of these CMOcs can be found in S2 Table. CMOcs were described in the context of the four phases of compression therapy: three CMOcs concerned process outcomes regarding initial compression therapy (treatment setting, start of initial compression therapy, and type of initial compression), one the onset of ECS therapy with selection of ECS class and type, one the implementation of assistive devices with selection and training of assistive devices, one the long-term maintenance of ECS therapy with individualized treatment duration for DVT patients and another one the provision of follow-up for CVD patients. Quantitative results regarding these main outcomes can be found in Tables 1, 2 and S2.

**Table 1:** Means, ranges, and resources clinical pathways deep venous thrombosis.

	**Limburg mean (%)**	**Range (%)**	**Source**	**North-Holland A mean (%)**	**Range (%)**	**North-Holland B mean (%)**	**Range (%)**	**Source**
**Initial compression therapy**								
**GP**	17	0–75	S_GP, L_	0	-	0	-	S_GP, NH_
No initial compression	20	0–50	S_GP, L_	-	-	-	-	-
MCB GP	40	-	S_GP, L_	-	-	-	-	-
MCB home care	40	-	S_GP, L_	-	-	-	-	-
**Internist**	83	-	S_GP, L_	100	-	100	-	S_GP, NH_
MCB home care	10	5–15	^1^^,^^2^ L	10	-	-	-	^1^^,^^2^ NH
TCH home care	4	-	^1^^,^^2^ L	4	-	-	-	^1^^,^^2^ NH
TCH self-reliant	86	-	^1^^,^^2^ L	86	-	-	-	^1^^,^^2^ NH
No initial compression	0	-	^1^^,^^2^ L	0	-	100	-	^1^^,^^2^ NH
	**Limburg mean (%)**	**Range (%)**	**Source**	**North-Holland mean (%)**		**Range (%)**		**Source**
**Onset of ECS therapy**								
Custom-made ECS	61	-	-	67		-		-
Ready-made ECS	39	7–95	S_MSS, L_	33		10–50		S_MSS,NH_
Class 2 ECS	30	0–90	S_MSS, L_	65		20–90		S_MSS,NH_
Class 3 ECS	68	0–100	S_MSS, L_	35		10–79		S_MSS,NH_
Other	2	0–10	S_MSS, L_	0		0–1		S_MSS,NH_
**Implementation of assistive devices**								
**Self-reliant without AD**	23	0–60	S_MSS, L_	50		0–80		S_MSS,NH_
**Self-reliant with AD without training**	56	-	-	35		-		-
Uncomplicated AD	53	15–80	S_MSS, L_	34		10–90		S_MSS,NH_
Complicated AD	3	0–10	S_MSS, L_	1		0–3		S_MSS,NH_
**Training by occupational therapist**	15	0–25	S_MSS, L_					
Via medical stocking supplier	15	0–25	S_MSS, L_	7		0–20		S_MSS,NH_
Additional training by home care	50	20–90	S_OCC,L_	43		0–71		S_OCC,L_
**Self-reliant with AD after training**	68	10–90	S_OCC,L_	66		36–95		S_OCC,L_
**To long term home care after training**	32	-	-	34		-		-
**Home care**	6	0–20	S_MSS, L_	8		1–20		S_MSS,NH_
**Directly to long term home care**	53	-	-	42		-		S_HN,NH_
Training by occupational therapist	-	-	-	58		0–100		S_HN,NH_
Training by home care	47	15–95	S_HN,L_	-		-		-
**Self-reliant with AD after training**	43	15–75	S_HN,L_	-		-		-
**To long term home care after training**	57	-	-	-		-		-
**Follow-up**								
**FU GP**	17	0–75	S_GP, L_					
**Standardized treatment duration**	100	-	^3^ Limburg	-		-		-
**Individualized treatment duration**	0	-	^3^ Limburg	-		-		-
**FU Internist**	83	-	-					
**Standardized treatment duration**	0	-	^1,2^ Limburg	100		-		^1^^,^^2^ NH
**Individualized treatment duration**	100	-	^1,2^ Limburg	0		-		^1^^,^^2^ NH

^1^: interview internist,

^2^: interview ER-nurse,

^3^: interview GPs

Abbreviations: L: Limburg, NH: North-Holland, GP: general practitioner, MCB: multilayer compression bandages, TCH: temporary compression hosiery, ECS: elastic compression stockings, AD: assistive device, S_GP, L_: survey GPs Limburg, S_GP,NH:_ survey GPs North-Holland, S_MSS, L_: survey medical stocking suppliers Limburg, S_MSS,NH_: survey medical stocking suppliers North-Holland, S_OCC,L_: survey occupational therapists Limburg, S_OCC,NH:_ survey occupational therapists North-Holland, S_HN,L_: survey home care nurses Limburg, S_HN,NH:_ survey home care nurses North-Holland

**Table 2:** Means, ranges, and resources clinical pathways chronic venous disease.

	Limburg mean (%)	Range %	Source	North-Holland Mean (%)	Range (%)	Source
**Initial compression therapy**						
**GP**	70	-	S_GP, L_	77	-	S_GP, NH_
No initial compression	10	0–50	S_GP, L_	8	0–50	S_GP, NH_
MCB GP	50	-	S_GP, L_	-	-	-
MCB home care	41	-	S_GP, L_	-	-	-
ACD self-reliant	-	-	-	44	5–80	S_HN,NH_
ACD not self-reliant	-	-	-	48	-	-
**Dermatologist**	30	5–75		23	0–100	
Patients treated conservatively with ECS therapy	100	-	Assumption, no data available	100	-	Assumption, no data available
MCB by home care	50	-	^1,2^ L	100	-	^1^^,^^2^ NH
MCB at outpatient clinic	50	-	^1,2^ L	0	-	^1^^,^^2^ NH
No initial compression	0	-	^1,2^ L	0	-	^1^^,^^2^ NH
**Onset of ECS therapy**						
Custom-made ECS	24	-	_—_	25	-	-
Ready-made ECS	76	70–85	S_MSS, L_	75	61–95	S_MSS,NH_
Class 2 ECS	77	20–95	S_MSS, L_	87	80–99	S_MSS,NH_
Class 3 ECS	23	5–80	S_MSS, L_	13	1–20	S_MSS,NH_
Others	0	0–1	S_MSS, L_	0	0–1	S_MSS,NH_
**Implementation of assistive devices**						
**Self-reliant without AD**	25	0–50	S_MSS, L_	33	0–65	S_MSS,NH_
**Self-reliant with AD without training**	45	-	-	53	-	-
With uncomplicated AD	38	20–80	S_MSS, L_	50	25–90	S_MSS,NH_
With complicated AD	7	0–20	S_MSS, L_	3	0–5	S_MSS,NH_
**Training by occupational therapist**						
Via medical stocking supplier	16	5–30	S_MSS, L_	7	0–20	S_MSS,NH_
Additional training by home care	50	10–90	S_OCC,L_	43	10–71	S_OCC,L_
**Self-reliant with AD after training**	68	10–90	S_OCC,L_	66	25–90	S_OCC,L_
**To long term home care after training**	32	-	S_OCC,L_	34	-	S_OCC,L_
**Home care**	14	0–35	S_MSS, L_	7	1–20	S_MSS,NH_
**Directly to long term home care**	53	-	-	42	-	S_HN,NH_
Training by occupational therapist	-	-	-	58	0–100	S_HN,NH_
Training home care	47	15–95	S_HN,L_	-	-	-
**Self-reliant with AD after training**	43	15–75	S_HN,L_	-	-	-
**To long term home care after training**	57	-	-	-	-	-
**Long-term maintenance of ECS therapy**						
**FU by GP**	70	-	S_GP, L_	77	-	S_GP, NH_
FU by default	40	-	S_GP, L_	20	-	S_GP, NH_
FU if problems exists	60	-	S_GP, L_	80	-	S_GP, NH_
**Dermatologist**	30	5–75	S_GP, L_	23	0–100	S_GP, NH_
FU by default	100	-	^1^	100	-	^1^
FU if problems exists	0	-	^1^	0	-	^1^

^1^ interview dermatologist,

^2^ interview nurse dermatology department

Abbreviations: L: Limburg, NH: North-Holland, GP: general practitioner, MCB: multilayer compression bandages, ACD: adjustable compression therapy, ECS: elastic compression stockings, AD: assistive device, S_GP, L_: survey GPs Limburg, S_GP, NH:_ survey GPs North-Holland, S_MSS, L_: survey medical stocking suppliers Limburg, S_MSS,NH_: survey medical stocking suppliers North-Holland, S_OCC,L_: survey occupational therapists Limburg, S_OCC,NH:_ survey occupational therapists North-Holland, S_HN,L_: survey home care nurses Limburg, S_HN,NH:_ survey home care nurses North-Holland

### Initial compression therapy

#### CMOc 1: Treatment setting

GPs generally have lower knowledge levels, exposure to compression therapy, and availability of diagnostic equipment compared to hospital specialists. These shortcomings are more relevant for DVT than for CVD. This influenced the willingness and commitment to treat DVT patients in primary care. As a result, most GPs referred DVT patients to secondary care whereas they treated most CVD patients in primary care. Moreover, in some elderly CVD patients, referral is not desired which triggered a reticent attitude to refer patients to secondary care.

#### CMOc 2: Start of initial compression therapy

Physicians’ knowledge levels and the extent to which they understood and valued the purposes of initial compression therapy influenced the willingness and commitment to prescribe it. Subsequently, variations exist in whether initial compression therapy was prescribed or omitted. In case physicians had insufficient knowledge, they relied on the local protocol or national guideline. If these provided unambiguous recommendations supporting the use of initial compression therapy, physicians’ commitment to implement these recommendations increased. Subsequently, the number of initial compression therapy prescriptions also increased.

Internists, GPs and dermatologists indicated that the extent to which patients were informed and had knowledge of the purposes of initial compression therapy influenced patients‘ motivation to accept and use initial compression therapy. Additionally, it increased patients’ self-confidence and self-efficacy which supported the treating physicians’ prescribing behavior. Some CVD patients had a negative attitude or perception towards compression therapy since they anticipate difficulties in applying the materials. This attitude triggered patients’ demotivation to accept and use compression therapy in general and physicians’ demotivation to prescribe it. Finally, the availability of trained staff to fit and demonstrate the use of initial compression therapy decreased stress levels and time constraints and improved the treating physicians’ motivation to prescribe it. Although treating physicians persistently perceived it to be difficult and time-consuming to manage initial compression therapy.

#### CMOc 3: Type of initial compression

Dermatologists (for CVD) and a minority of internists (for DVT) had sufficient knowledge regarding initial compression therapy types and their effects on maintaining self-reliance. This drove the willingness and commitment to prescribe the most suitable initial compression therapy. As a result, the number of self-reliant patients increased. Most internists and GPs lacked this knowledge treating DVT patients. Therefore, they relied on- and committed to the local protocol or national guideline. These protocols and guidelines provided diverse recommendations and guidance regarding the preferred initial compression therapy. This ambiguity in recommendations resulted in a diversity of treatment processes influencing the patient’s opportunity to maintain self-reliance. Some GPs in region North-Holland were more focused on patient-based treatments for CVD patients since they experienced benefits for patients. In this case, the use of adjustable compression devices was supported by local arrangements. In these arrangements it was agreed that management and reimbursement were covered by the home care organization. As a result, GPs were motivated and self-confident to prescribe adjustable compression devices which allowed approximately half of the patients to maintain self-reliance.

With increased availability of trained staff physicians’ motivation to make use of the staff’s capabilities to instruct and demonstrate how to apply the initial compression therapy increased. As a result, patients were better informed and their self-efficacy increased leading to a higher likelihood for patients to remain self-reliant. Additionally, patients’ knowledge, aims, and desires regarding initial compression therapy were identified as conditional to achieve optimal patient involvement in the decision-making process. As a result, patients’ likelihood to receive the most appropriate initial compression therapy increased with enhanced possibilities for self-reliance.

### Onset of ECS therapy

#### CMOc 4: Selection of ECS class and type

Depending on the physician’s knowledge levels in determining the treatment indication and ECS characteristics (class and type) the willingness, confidence, and commitment to include the ECS characteristics in the referral to the medical stocking supplier varied. If physicians had insufficient knowledge they committed to the local protocol or the national guideline (which in some cases were not up to date) or omitted including the ECS characteristics in the referral. This increased the number of ECS referrals that were not evidence-based or incomplete.

If referrals were complete, the medical stocking supplier generally delivered the ECS as indicated by the treating physician based on confidence and commitment to approach and instructions. In case referrals were incomplete, medical stocking suppliers decided to select the ECS characteristics themselves. Medical stocking suppliers are bound to financial constraints as they receive a fixed cluster fee per patient which is perceived to be insufficient. In some cases these constraints triggered a reticent attitude toward the implementation of more expensive ECS classes and types resulting in a decrease in evidence-based prescriptions.

The availability of accessible interdisciplinary communication and understanding of responsibilities as well as mutual expectations affected the willingness and motivation to improve interdisciplinary processes. Consequently, information transfers enhanced and the delivery of evidence-based ECS prescriptions increased.

### Implementation of assistive devices

#### CMOc 5: Selection and training of assistive devices

The extent to which patients desired to be self-reliant to apply the ECS in combination with the physical and cognitive abilities they possess influenced the patient’s motivation to use an assistive device, their involvement in the decision-making process, and their willingness to use their abilities. As a result, the likelihood of patients maintaining their self-reliance varied. The presence of an informal caregiver for patients lacking these abilities provided the opportunity to involve the caregiver in the selection and training process to support the patient. In turn, the number of patients requiring additional training or long-term home care decreased.

Furthermore, the extent to which medical stocking suppliers experienced time and financial constraints varied. As an effect, their motivation and willingness to invest time in the selection and training of patients to use an assistive device varied. If training duration was based on the patient’s needs, their possibility to maintain self-reliance increased. If medical stocking suppliers experienced financial constraints, a reticent attitude to invest time and costs was triggered. As a result, the number of patients referred for additional training or long-term home care increased.

If patients were referred for additional training, differences in levels of training- and knowledge regarding assistive devices existed between occupational therapists and home care nurses. Occupational therapists generally applied patient-based methods to select an assistive device and training frequency and duration were based on the patient’s needs. Most home care nurses selected an assistive device based on personal experience and training duration depending on the available time left during the visit. If a patient-based method was implemented, the patient’s likelihood to maintain self-reliance with the use of an assistive device increased. Moreover, medical stocking suppliers and occupational therapists had well-established interdisciplinary communication whereas communication between medical stocking suppliers and home care nurses was spare or absent. This affected the ability, willingness, and motivation to collaborate and improve interdisciplinary processes and the extent to which patient information was transferred. In return, it affected the patient’s opportunities to maintain self-reliance.

Finally, home care nurses perceived it to be more difficult to encourage patients to be self-reliant if they were already used to home care because patients were anxious to lose these services. This led to a lack of motivation in patients and decreased the patient’s likelihood to be self-reliant with an assistive device.

### Long-term maintenance of ECS therapy

#### CMOc 6: Individualized duration of ECS therapy for DVT patients

Internists’- and GPs’ knowledge levels of individually tailored treatment duration and how to assess eligibility for shortened treatment duration influenced the extent to which physicians could commit to an individualized approach. This resulted in two outcomes: some physicians advised a standardized treatment duration of two years whereas others advised an individualized treatment duration.

In case physicians lacked knowledge they relied on the local protocol or national guideline which showed ambiguity in their recommendations. This led to different treatment advice. Furthermore, depending on whether the GPs and internists felt responsible for follow-up, their motivation and willingness to invest time and effort in shaping this follow-up varied. Consequently, some physicians had a reticent attitude to implement individualized follow-up. Finally, individualized treatment duration cannot work without interdisciplinary cooperation and communication between all involved health professionals and patients. Currently, some patients received automated annual calls from the medical stocking supplier although their treatment was already terminated. In some cases this resulted in patients resuming ECS therapy without interference from the treating physician.

#### CMOc 7: Performing follow-up for CVD patients

The degree to which physicians value the need for follow-up and the disease severity determined physicians’ motivation and willingness to invest time in follow-up appointments. GPs often do not value this need when other health care professionals (i.e., edema therapists, home care nurses, or medical stocking suppliers) are involved in monitoring the process and contacting the GP when applicable. This induced a lack of involvement to perform follow-up. However, home care nurses and medical stocking suppliers experience these follow-up appointments as essential since they often encounter difficulties with patients who have additional questions or lack adherence to therapy that they cannot resolve.

### Overview of overarching CMOcs

**Fig 1:**
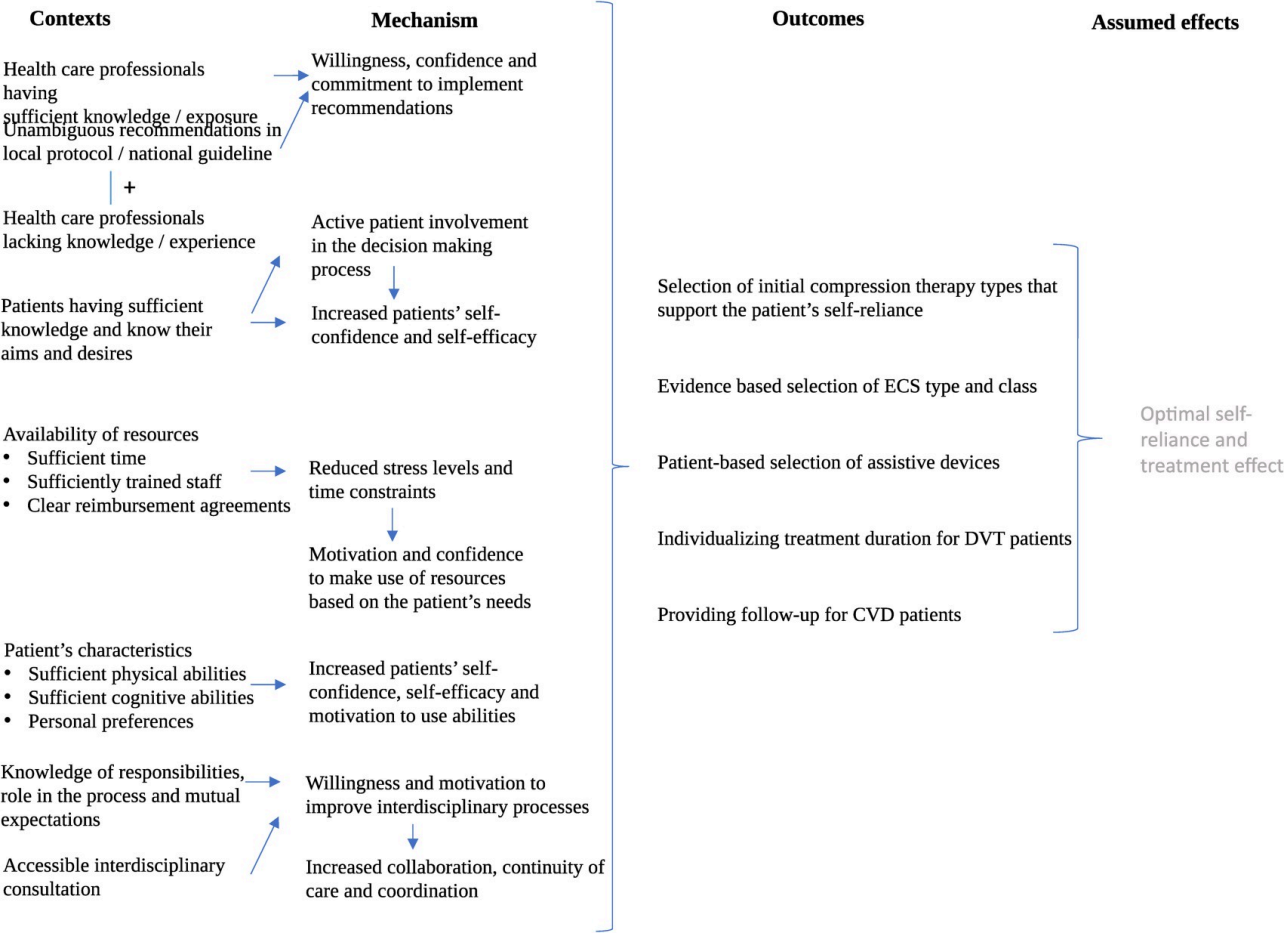
Graphical overview of the overarching CMOcs resulting in desirable outcomes. Legend. Abbreviations: ECS: elastic compression therapy, DVT: deep venous thrombosis, CVD: chronic venous disease.

In our CMOcs, we identified several overarching contextual factors and mechanisms resulting in desirable outcome as presented in Fig 1. Based on these overarching CMOcs, we ultimately identified five targets for improvement strategies: 1. Increase all health care professionals’ knowledge of compression therapy, 2. Provide unambiguous recommendations in guidelines and protocols, 3. Involve patients (and if applicable the informal caregivers) in the decision-making process, 4. Provide access to resources (time, trained staff, and clear reimbursement agreements), and 5. Promote interdisciplinary consultation.

## Discussion

This study used a realist evaluation to understand what works and does not work, for whom, and under which circumstances in the organization of compression therapy for DVT and CVD patients. Our results indicate a lack of knowledge among treating physicians regarding compression therapy in general, leading to incomplete referrals to medical stocking suppliers. For example, the level of class 3 ECS prescriptions for DVT patients was below what was to be expected from evidence [1, 2, 11, 22]. Increasing knowledge most likely will result in more complete referrals. Consequently, as most medical stocking suppliers commit to the treating physicians referrals, financial incentives currently influencing the medical stocking supplier’s choice for ECS-type will likely become less relevant. To further minimize the role of financial incentives, we suggest that the financial reward system for medical stocking suppliers needs to be reformed. Furthermore, the lack of knowledge among health care professionals not only resulted in suboptimal selection of compression therapy but also in suboptimal provision of assistive devices to promote patients’ self-reliance.

Knowledge improvement will also likely increase the use of an individualized risk-based treatment duration and thus prevent unnecessary long treatment duration without benefits for the patients [2]. The prerequisite for successful implementation of individualized ECS treatment duration needs broad consensus on who is responsible for this type of management and follow-up. Additionally, it is important to communicate treatment duration between treating physicians, the medical stocking supplier, and the homecare organization.

However, health care professionals acknowledged that this specific knowledge rapidly declines since most stakeholders were working in broad disciplines covering a large variety of diseases. Therefore, detailed protocols and guidelines are mandatory to provide sustainable practical support to guide these decisions. It is shown that protocols and guidelines are especially useful if they pay attention to contexts [16]. In case of insufficient knowledge, our CMOcs showed that professionals used local protocols or the national guideline (GPs) by default. Currently there is ambiguity amongst protocols and because of different recommendations a variety of outcomes has been observed. Especially for the recommended initial compression therapy types and the level to which self-reliance is considered to inform the treatment decision. Making protocols unambiguous prioritizing temporary compression hosiery for DVT [2, 23] and adjustable compression devices for CVD [24, 25] will likely increase the number of self-reliant patients at this stage.

Furthermore, the role of the patient and (if present) the informal caregiver in the various stages of the decision-making process needs to be enhanced. This may be achieved by improved information transfer, shared decision making, and providing motivation and support to better define patients’ aims and desires. The involvement of patients in the decision-making process is of major importance since higher levels of involvement are positively correlated with clinical outcomes like adherence to therapy and coordination of care [26–28]. Furthermore, it is important to consider patients’ characteristics (physical and cognitive abilities and personal preferences) in shared decision making.

The availability of sufficient resources is also important role for optimizing outcomes as it facilitates a patient-based approach to select compression materials and assistive devices. For this, clear reimbursement agreements, sufficient time and trained staff, and a sufficient reward system is necessary. These resources facilitated a patient-based approach to select compression materials and assistive devices. Finally, for all improvement opportunities, it is pivotal to create clarity among involved professionals concerning their specific role in the process and to improve knowledge of mutual expectations and responsibilities. This is supposed to increase the motivation to improve interdisciplinary processes resulting in overall optimization of outcomes [29–33].

Although this study did not primarily focus on patient’s adherence, which is one of the most important determinants for effectiveness of compression therapy, we expect that the proposed process improvement will be associated with increased patients’ adherence. However, further research on long-term adherence and health care outcomes after implementation of the improvements is necessary. Multidimensional interventions to improve adherence were found the show promising results [34], although further investigations with high-quality trials are required.

Some weaknesses of our study have to be considered. Although we identified a large variety of CMOcs based on interview data and stakeholder meetings, direct observations of the care delivered were not possible due to the COVID-19 pandemic. It is therefore possible that some contextual factors and mechanisms were missed since health care professionals were not aware of these factors and did not mention them. Furthermore, although we diligently approached stakeholder groups and received adequate response rates, response rates for occupational therapists and home care nurses in Limburg were lower. This might have biased our results. The main strength of this study is that it provides not only a detailed insight into how ECS therapy is organized, but also on how outcomes are influenced by contexts and mechanisms, resulting in the identification of targets for improvement. As previously observed in implementation science, many studies lack this attention to theory development preceding the implementation of complex interventions which is thought to limit implementation [35–37]. Finally, although the CMOcs are designed to be specific to DVT- and CVD patients, the configurations and targets for improvement are likely to be transferable to other patient groups using compression therapy such as patients with lymphedema. The degree to which the CMOcs are transferable to other countries depends on how compression therapy is organized locally. However, the approach we used to identify targets for improvement is generalizable and could be used to evaluate and improve compression therapy in other counties as well.

## Conclusion

This realist evaluation gives a detailed insight into what works (and does not work), for whom, and under which circumstances for both patients and health care professionals in compression care. We identified five targets for improvement of compression care: increase health care professionals’ knowledge of compression therapy, increase the availability of unambiguous protocols and guidelines, increase the involvement of patients in the decision making process, increase the accessibility of resources, and increase interdisciplinary consultation.

## Supporting information

S1 File Interview template. (DOCX)

S2 File Survey questions. (DOCX)

S1 Table **Table 1**: Distribution of interviewees per stakeholder group. Legend: Adopted from Schreurs RHP, Joore MA, Ten Cate H, Ten Cate-Hoek AJ. Using the Functional Resonance Analysis Method to explore how elastic compression therapy is organised and could be improved from a multistakeholder perspective. BMJ open. 2021 Oct 12;11(10):e048331. PubMed PMID: 34642192. Pubmed Central PMCID: PMC8513256. Epub 2021/10/14. eng. **Table 2**: Response rate per professional group (survey).
(DOCX)

S2 Table **Table 1**: Referral behavior. Legend: Abbreviations L: Limburg, NH: North-Holland, GP: general practitioner, DVT: deep venous thrombosis, CVD: chronic venous disease. **Table 2**: Start of initial compression therapy. Legend: Abbreviations L: Limburg, NH-A: North-Holland location A, NH-B: North-Holland location B, GP: general practitioner, DVT: deep venous thrombosis, CVD: chronic venous disease. **Table 3**: Type of initial compression. Legend: Abbreviations L: Limburg, NH-A: North-Holland location A, GP: general practitioner, DVT: deep venous thrombosis, CVD: chronic venous disease, ACD: adjustable compression devices, TCH: temporary compression hosiery, MCB: multilayer compression bandages. **Table 4**: Information and patient transfer. Legend: Abbreviations L: Limburg, NH: North-Holland, GP: general practitioner, DVT: deep venous thrombosis, CVD: chronic venous disease, ECS: elastic compression stockings. **Table 5**: Selection and training of assistive devices. Legend: * Context factors related to patients who require additional training. Abbreviations L: Limburg, NH: North-Holland, AD: assistive device, DVT: deep venous thrombosis, CVD: chronic venous disease. **Table 6**: Individualized duration of ECS therapy for DVT patients. Legend: Abbreviations L: Limburg, NH: North-Holland, NH-B: North-Holland location B, GP: general practitioner, DVT: deep venous thrombosis, ECS: elastic compression stockings. **Table 7**: Performing follow-up for CVD patients. Legend: Abbreviations L: Limburg, NH: North-Holland, GP: general practitioner, CVD: chronic venous disease.
(DOCX)
