# Supplementary material for: A realist evaluation to identify targets to improve the organization of compression therapy for deep venous thrombosis- and chronic venous disease patients
Source: PLoS One. 2022 Aug 8;17(8):e0272566. doi: 10.1371/journal.pone.0272566 (PMC9359574; doi:10.1371/journal.pone.0272566)
Supplement: S1 File — (DOCX) [file pone.0272566.s001.docx]

**Supporting information 1: interview template**

Main questions for health care professionals

Can you tell me what your role in compression therapy exactly is?

Do you treat/assist patients with deep venous thrombosis, chronic venous disease, or both?

If you see compression therapy as a whole, which factors are important outcomes to patient care?

You mentioned (outcome named in the previous question) as an important outcome. Is there variation in outcomes in your practice or is the outcome always the same? How can health care professionals empower patients to achieve this outcome? Are there any treatment decisions that influence the patient’s chances to achieve desirable outcomes?

Practically, it was our impression that there is a substantial variation in how ECS therapy is organized in current care, with various effects on outcomes. Do you acknowledge this variation within the process? And within your discipline/your work?

If there is variation, how can you explain this variation? What factors influenced you had outcome x for patient 1 and outcome y for patient 2? How does this variation affect your work/how do you deal with this variation? How do you think other health care professionals in your discipline deal with this variation? Does this affect outcomes? Can you identify specific targets leading to desirable/undesirable outcomes?

If you could change something to make your current work more efficient and achieve higher desirable outcomes, what would you change and why?

Main questions for patients

Are you currently being treated with compression therapy? For which disease are you being treated?

Have you been treated before with other types of compression therapy (for example, multilayer compression bandages, temporary compression hosieries, or adjustable compression devices)?

What are important factors/outcomes for you during treatment? Can you give an example of (outcome named in the previous question)? Did these important factors change during the process? Did you discuss these factors with your treating physician? If yes, did they support you in achieving these outcomes and how exactly did they help you?

Do you apply and remove the compression materials self-reliant or do you need home care assistance to assist you? If the patient required home care assistance, is it a problem for you that you require home care assistance? If yes, do you think health care professionals maximally empowered you to maintain self-reliance? If not, what do you think you need to function self-reliant?

Do you use your compression materials? If not, why not? If yes, do you use them daily? If not, why not?
