# Supplementary material for: A realist evaluation to identify targets to improve the organization of compression therapy for deep venous thrombosis- and chronic venous disease patients
Source: PLoS One. 2022 Aug 8;17(8):e0272566. doi: 10.1371/journal.pone.0272566 (PMC9359574; doi:10.1371/journal.pone.0272566)
Supplement: S2 File — (DOCX) [file pone.0272566.s002.docx]

**Supporting information 2: Survey questions.**

Only the questions in bold were used in the current paper.

- 1. **Survey medical stocking suppliers**

In this survey, the term CVD refers to patients with a stage C4 and C5 CVD according to the CEAP (Clinical-Etiological-Anatomical-Pathophysiological) classification system.

**1. Are you currently working for a company chain or an independent company? [MC]**

2. How long does an intake consult take? [slider 0-90 minutes]

**3. What percentage of CVD-patients is treated with ready-made ECS? [slider 0-100%]**

**4. What percentage of CVD-patients is treated with the following classes of ECS: [Must total 100%]**

- **Class 2**
- **Class 3**
- **Other**

**5. Which factors affect the chosen type (custom-made or ready-made), and compression strength for CVD-patients? [OQ]**

**6. What percentage of DVT-patients is treated with confection ECS? [slider 0-100%]**

**7. What percentage of DVT-patients is treated with the following classes of ECS: [Must total 100%]**

- **Class 2**
- **Class 3**
- **Other**

**8. Which factors affect the chosen type (custom-made or ready-made), and compression strength for DVT-patients? [OQ]**

9. What is the average delivery time for ready-made ECS? [slider 0-25 days]

10. What is the average delivery time for custom-made ECS? [slider 0-25 days]

11. How long does a fitting consult take in general? [slider 0-45 minutes]

12. What percentage of patients receive a fitting consult by default? [slider 0-100%]

14. What percentage of patients receive a physical follow-up appointment after one year? [slider 0-100%]

15. What percentage of patients receive a physical follow-up appointment in the subsequent years? [slider 0-100%]

**16. After delivery of the definitive ECS, what percentage of CVD-patients applies the following situations: [Must total 100%]**

- **The patient is self-reliant without an assistive device**
- **The medical stocking supplier implements an uncomplicated assistive device (i.e. resistance reducing device or a cuff and cone)**
- **The medical stocking supplier implements a complicated assistive device (i.e. frames or arm-extending devices)**
- **The patient is referred to the occupational therapist**
- **The patient is advised to contact the home care organization**

**17. How do you explain these percentages? Which factors affect the patient’s course? [OQ]**

**18. After delivery of the definitive ECS, what percentage of DVT-patients applies the following situations: [Must total 100%]**

- **The patient is self-reliant without an assistive device**
- **The medical stocking supplier implements an uncomplicated assistive device (i.e. resistance reducing device or a cuff and cone)**
- **The medical stocking supplier implements a complicated assistive device (i.e. frames or arm-extending devices)**
- **The patient is referred to the occupational therapist**
- **The patient is advised to contact the home care organization**

**19. How do you explain these percentages? Which factors affect the patient’s course? [OQ]**

Abbreviations: CVD: chronic venous insufficiency, DVT: deep venous thrombosis, ECS: elastic compression stockings, MC: multiple-choice, OQ: open question

**1.2 Survey occupational therapists**

**1. Are you currently working for a company chain or an independent company? [OQ]**

2. How many hours do you spend, on average, to select and implement an assistive device with the patient (including the intake consult)? [slider 0-10 hours]

**3. What percentage of patients ultimately succeeds to be self-reliant after training? [slider 0-100%]**

**4. Which factors affect the chosen assistive device? [OQ]**

**5. Which factors affect the patient’s possibilities to be self-reliant with the use of an assistive device? [OQ]**

4. For what percentage of patients do you ask the home care organization to provide additional training? [slider 0-100%]

5. For how long do you ask the home care organization to provide additional training on average in these patients? [slider 0-30 days]

**6. For what percentage of patients do you implement the following assistive devices for training: [Must total 100%]**

- **Resistance reducing devices**
- **Cuffs and cones**
- **Frames and arm-extending devices**
- **Elastic compression stocking pistol**

**7. How do you choose and select the appropriate assistive device? Which factors affect your choice? [OQ]**

Abbreviations: OQ: open question

**1.3 Survey home care nurses Limburg**

**1. What percentage of patients, referred back to home care after the definitive elastic compression stocking is delivered, is referred to the specialized home care nurse (ergocoach) to train an assistive device? [slider 0-100%]**

**2. How do you select patients for referral? Which factors affect your choice? [OQ]**

3. How many hours do you spend, on average, to select and implement an assistive device with the patient (including the intake consult)? [slider 0-5 hours]

4. For what time frame do you ask regular home care nurses, on average, to provide additional training in these patients? [slider 1-6 weeks]

**5. What percentage of patients ultimately succeeds to be self-reliant after training? [slider 0-100%]**

**6. Which factors affect the chance of successful implementation of an assistive device? [OQ]**

**7. For what percentage of patients do you use the following assistive devices for training: [Must total 100%]**

- **Resistance reducing devices**
- **Cuffs and cones**
- **Frames and arm-extending devices**
- **ECS pistol**

**8. How do you choose and select the appropriate assistive device? Which factors affect your choice? [OQ]**

**1.4 Survey home care nurses North-Holland**

1. How long does an ACD fitting consult takes on average (including instruction)? [slider 0-120 minutes]

**2. What percentage of patients can self-reliantly apply and adjust the ACD after the instruction consult in the initial compression phase? [slider 0-100%]**

**3. Which factors affect the patient's possibilities to self-reliantly apply and adjust the ACD? [OQ]**

4. What percentage of patients require a footwrap in addition to the adjustable compression device? [slider 0-100%]

5. What percentage of patients require another adjustable compression device as type A? [slider 0-100%]

6. How long do you generally apply ACD before the patient is instructed to call the medical stocking supplier to fit the definitive ECS? [slider 1-9 weeks]

**7. What percentage of patients, referred back to home care after the definitive elastic compression stocking is delivered, is referred to the occupational therapist to train an assistive device? [slider 0-100%]**

**8. How do you select patients for referral to the occupational therapist? Which factors affect your choice? [OQ]**

Abbreviations: ACD: adjustable compression device, ECS: elastic compression stocking, OQ: open question

**1.5 Survey general practitioners**

In this survey, the term CVD refers to patients with a stage C4 to C5 according to the CEAP (Clinical-Etiological-Anatomical-Pathophysiological) classification system.

Questions regarding DVT:

**1. Do you treat DVT-patients independently at your general practice? Yes/no**

If not: **1a. What are the reasons you do not treat these patients? [OQ]**

If yes:

**1b. What percentage of patients do you refer to secondary care? [slider 0-100%]**

**1c. Which factors affect whether you refer a patient to secondary care for the treatment of DVT? [OQ]**

**1d. For what percentage of DVT-patients do you prescribe initial compression therapy? [slider 0-100%]**

**1e. Which factors affect whether you prescribe initial compression therapy? [OQ]**

**1f. Do you ever use other types of initial compression therapy than multilayer compression bandages? Yes/no**

Questions regarding CVD:

**2. What percentage of CVD patients are referred to secondary care? [slider 0-100%]**

**3. Which factors affect whether you refer the patient to secondary care? [OQ]**

**4. If you decide to treat the patient at the general practice, for what percentage of CVD patients do you prescribe initial compression therapy? [slider 0-100%]**

**5. Which factors affect whether you prescribe initial compression therapy? [OQ]**

**6. Only for GPs in Limburg: Do you ever use other types of initial compression therapy than multilayer compression bandages? Yes/no.**

**If yes**

**6a. What other types of initial compression therapy do you prescribe? [OQ]**

**6b. For what percentage of patients do you prescribe other types of initial compression therapy? [slider 0-100%]**

**7. Only for GPs in North-Holland: Do you ever use other types of initial compression therapy than adjustable compression devices? Yes/no.**

**If yes**

**7a. What other types of initial compression therapy do you prescribe? [OQ]**

**7b. For what percentage of patients do you prescribe other types of initial compression therapy? [slider 0-100%]**

**8. What percentage of CVD patients receive a follow-up appointment by default?**

**9. Which factors affect whether you plan a follow-up appointment?**

Abbreviations: CVD: chronic venous insufficiency, DVT: deep venous thrombosis, GP: general practitioner, OQ: open question
