# Supplementary material for: A realist evaluation to identify targets to improve the organization of compression therapy for deep venous thrombosis- and chronic venous disease patients
Source: PLoS One. 2022 Aug 8;17(8):e0272566. doi: 10.1371/journal.pone.0272566 (PMC9359574; doi:10.1371/journal.pone.0272566)
Supplement: S1 Table — Table 1: Distribution of interviewees per stakeholder group. Legend: Adopted from Schreurs RHP, Joore MA, Ten Cate H, Ten Cate-Hoek AJ. Using the Functional Resonance Analysis Method to explore how elastic compression therapy is organised and could be improved from a multistakeholder perspective. BMJ open. 2021 Oct 12;11(10):e048331. PubMed PMID: 34642192. Pubmed Central PMCID: PMC8513256. Epub 2021/10/14. eng. Table 2: Response rate per professional group (survey). (DOCX) [file pone.0272566.s003.docx]

**Supporting information 3:**

**Table 1: Distribution of interviewees per stakeholder group**

| **Interviewees** | **Limburg (15)** | **North-Holland (15)** |
| --- | --- | --- |
| General practitioners | 2 | 2 |
| Internists | 2 | 3 |
| Dermatologist | 1 | 1 |
| ER nurses | 2 | 1 |
| Doctor’s assistant dermatology | n/a | 1 |
| Nurse dermatology | 1 | n/a |
| Medical stocking suppliers | 2 | 1 |
| Home care nurses | 2 | 2 |
| Occupational therapists | 1 | 1 |
| Deep venous thrombosis patients | 1 | 1 |
| Chronic venous insufficiency patients | 1 | 2 |

Adopted from Schreurs RHP, Joore MA, Ten Cate H, Ten Cate-Hoek AJ. Using the Functional Resonance Analysis Method to explore how elastic compression therapy is organised and could be improved from a multistakeholder perspective. BMJ open. 2021 Oct 12;11(10):e048331. PubMed PMID: 34642192. Pubmed Central PMCID: PMC8513256. Epub 2021/10/14. eng.

**Table 2: Response rate per professional group (survey)**

|  | **Limburg** | | | | **North-Holland** | | | |
| --- | --- | --- | --- | --- | --- | --- | --- | --- |
| **Professionals** | **Invited practices (n)** | **Practices agreed to participate (n)** | **Invited professionals (n)** | **Response rate (%)** | **Invited practices (n)** | **Practices agreed to participate (n)** | **Invited professionals (n)** | **Response rate (%)** |
| **Occupational therapists** | 7 | 6 | 34 | 47 | 7 | 6 | 21 | 67 |
| **Medical stocking suppliers** | 5 | 5 | 8 | 88 | 4 | 5 | 5 | 100 |
| **Home care nurses** | 1 | 1 | 24 | 67 | 1 | 1 | 40 | 85 |
| **General practitioners** | 18 | 12 | 12 | 100 | 15 | 10 | 10 | 100 |
