# Supplementary material for: A realist evaluation to identify targets to improve the organization of compression therapy for deep venous thrombosis- and chronic venous disease patients
Source: PLoS One. 2022 Aug 8;17(8):e0272566. doi: 10.1371/journal.pone.0272566 (PMC9359574; doi:10.1371/journal.pone.0272566)
Supplement: S2 Table — Table 1: Referral behavior. Legend: Abbreviations L: Limburg, NH: North-Holland, GP: general practitioner, DVT: deep venous thrombosis, CVD: chronic venous disease. Table 2: Start of initial compression therapy. Legend: Abbreviations L: Limburg, NH-A: North-Holland location A, NH-B: North-Holland location B, GP: general practitioner, DVT: deep venous thrombosis, CVD: chronic venous disease. Table 3: Type of initial compression. Legend: Abbreviations L: Limburg, NH-A: North-Holland location A, GP: general practitioner, DVT: deep venous thrombosis, CVD: chronic venous disease, ACD: adjustable compression devices, TCH: temporary compression hosiery, MCB: multilayer compression bandages. Table 4: Information and patient transfer. Legend: Abbreviations L: Limburg, NH: North-Holland, GP: general practitioner, DVT: deep venous thrombosis, CVD: chronic venous disease, ECS: elastic compression stockings. Table 5: Selection and training of assistive devices. Legend: * Context factors related to patients who require additional training. Abbreviations L: Limburg, NH: North-Holland, AD: assistive device, DVT: deep venous thrombosis, CVD: chronic venous disease. Table 6: Individualized duration of ECS therapy for DVT patients. Legend: Abbreviations L: Limburg, NH: North-Holland, NH-B: North-Holland location B, GP: general practitioner, DVT: deep venous thrombosis, ECS: elastic compression stockings. Table 7: Performing follow-up for CVD patients. Legend: Abbreviations L: Limburg, NH: North-Holland, GP: general practitioner, CVD: chronic venous disease. (DOCX) [file pone.0272566.s004.docx]

**Supporting information 4: CMOcs**

**Initial compression therapy**

**Table 1:** **Referral behavior**

| **Context (+)** | **Mechanism (=)** | **Distal outcome** |
| --- | --- | --- |
| **In favor of referral** |  | **DVT**  *Primary care*  L: 11%, NH: 0%  *Secondary care*  L: 89%, NH: 100%  **CVD**  *Primary care*  L: 70%, NH: 76%  *Secondary care*  L: 30%, NH: 24% |
| **DVT** |  |  |
| Lack of knowledge/exposure: Most GPs have limited knowledge regarding the treatment of DVT and limited exposure to DVT-patients | Lack of confidence, and commitment to treat DVT-patients. |  |
| Lack of diagnostic equipment: Most GPs lacked an ultrasound (L, NH-A), or even the possibility to direct order a diagnostic ultrasound without interference of the internist (NH-B). | Lack of motivation and commitment to use resources |  |
| **DVT and CVD** |  |  |
| Patient characteristics: Younger and patient’s with a higher risk for complications (e.g. with multiple comorbidities, known bleeding disorders, or recurrent DVT’s) are more likely to be referred to exclude underlying pathology | Risk management: In younger and complicated patients, GPs do not tolerate any risk of missing an underlying disease |  |
| **In favor of treatment in primary care** |  |  |
| **DVT** |  |  |
| Knowledge/exposure: Specialized GPs have sufficient knowledge regarding the treatment of DVT and sufficient exposure to DVT-patients | Willingness and commitment to treat DVT-patients |  |
| **DVT and CVD** |  |  |
| Patient characteristics: In some elderly interference of secondary care is not desired by the GP or the patient | Reticent attitude regarding referrals in some elderly |  |
| **CVD** |  |  |
| Knowledge/exposure: All GPs have sufficient knowledge regarding the treatment of CVD and sufficient exposure to CVD-patients | Willingness and commitment to treat CVD-patients |  |
| Availability of diagnostic equipment: GPs have the appropriate equipment to diagnose CVD | Motivation and commitment to make use of resources |  |

Abbreviations L: Limburg, NH: North-Holland, GP: general practitioner, DVT: deep venous thrombosis, CVD: chronic venous disease

**Table 2:** **Start of initial compression therapy**

| **Context (+)** | **Mechanism (=)** | **Proximal outcome** | **Distal outcome** |
| --- | --- | --- | --- |
| **Enhancing** |  |  | **DVT**  *Initial compression therapy:*  GP L: 90 % Internist L/NH-A: 100%, NH-B:0%  *No initial compression therapy:*  GP L: 10%  Internist L/NH-A: 0%, NH-B: 100%  **CVD**  *Initial compression therapy:*  GP L: 90%, NH: 88%  Dermatologist L+NH: 100%  *No initial compression therapy:* GP L: 10%, NH: 12%  Dermatologist L+NH: 0% |
| **DVT and CVD** |  |  |  |
| Knowledge levels: Most internists (L+NH-A), dermatologists (L+NH), and GPs (L+NH) have sufficient knowledge to treat DVT patients, they understand and value the underlying purpose of initial compression therapy | Willingness, confidence and commitment to prescribe initial compression therapy | Increased number of initial compression therapy prescription |  |
| Lack of knowledge: Some internists (L+NH-A), dermatologists (L), and GPs (L+NH) lack knowledge regarding initial compression therapy. The local protocol provides unambiguous recommendations for using initial compression therapy | Commitment to implement recommendations | Increased number of initial compression therapy prescription |  |
| Patient’s knowledge: Sufficiently informed patients who want to prevent complications or reduce symptoms | Patients’ motivation to accept and use initial compression therapy  Increased self-efficacy and self-confidence | Increased number of initial compression therapy prescriptions |  |
| Trained staff: Availability of trained staff to fit and demonstrate initial compression therapy | Physician’s motivation to use staff’s capabilities  Professionals experiencing less stress and less time constraints | Increased number of initial compression therapy prescriptions |  |
| **Inhibiting** |  |  |  |
| **DVT** |  |  |  |
| Lack of knowledge: Internists are not aware, or lack knowledge, regarding the effectiveness of initial compression therapy for DVT (NH-B). The local protocol does not provide recommendations for using initial compression therapy | Lack of willingness and commitment to prescribe initial compression therapy | Omission of initial compression therapy |  |
| Lack of trained staff: Available staff is not trained to fit and demonstrate the initial compression therapy for DVT (NH-B). If no trained staff is available, it is perceived as difficult and inefficient to arrange care. | Reticent attitude regarding the use of initial compression therapy | Omission of initial compression therapy |  |
| **CVD** |  |  |  |
| Patient characteristics: Some patients expect difficulties in applying compression therapy in general, i.e. due to a lack of strength. Furthermore, compression therapy generally has a negative image to most patients | Patient’s demotivation, reticent attitude | Decreased number of initial compression therapy prescription |  |

Abbreviations L: Limburg, NH-A: North-Holland location A, NH-B: North-Holland location B, GP: general practitioner, DVT: deep venous thrombosis, CVD: chronic venous disease

**Table 3:** **Type of initial compression**

| **Context (+)** | **Mechanism (=)** | **Proximal outcome** | **Distal outcome** |
| --- | --- | --- | --- |
| **Enhancing** |  |  | **DVT**  *TCH self-reliant:*  L: 77%, NH: 86%  *TCH home care*  L: 4%, NH: 4%  *MCB not self-reliant*  L: 19%, NH:10%  *No initial compression therapy:*  L: 1%, NH-A: 0%, NH-B: 100%  **CVD**  *MCB not self-reliant*  L: 94%, NH: 24%  *ACD self-reliant*  NH: 32%  *ACD not self-reliant*  NH: 35 %  *No compression*  L: 7 %, NH: 9 % |
| **DVT** |  |  |  |
| Lack of knowledge: Some internists lack knowledge regarding the different types of initial compression therapy (L+NH-A). The local protocol provides unambiguous recommendations as regards preference for TCH | Commitment to prescribe the recommended initial compression therapy | TCH is prescribed, the largest part of patients remains self-reliant |  |
| **DVT and CVD** |  |  |  |
| Knowledge: Some internists (for DVT) and dermatologists (for CVD) know the different types of initial compression therapy, their effectiveness, and their effects on the ability of patients to maintain their self-reliance (L+NH-A) | Willingness and commitment to prescribe the most suitable initial compression therapy | Patients receive the most suitable type of initial compression therapy, increased number of self-reliant patients |  |
| Trained staff: At the ER and the dermatology department, trained staff is available to fit and demonstrate the initial compression therapy (L + NH-A) | Motivation to make use of staff’s capacities and experience  Professionals experiencing less stress and less time constraints | Patients are sufficiently instructed to use the initial compression therapy, increased number of self-reliant patients |  |
| Patient characteristics: Patients who have the sufficient strength and cognitive abilities to apply and remove the initial compression therapy self-reliantly | Motivation to make use of their abilities | The likelihood of the patient maintaining self-reliance increases |  |
| Patient’s knowledge: Sufficiently informed patients who know their aims and desires regarding initial compression therapy | Active patient involvement in the decision-making process  Increased self-efficacy and self-confidence | Increased likelihood that the patient receives the most suitable type of initial compression therapy |  |
| **CVD** |  |  |  |
| GPs (NH) have knowledge regarding the use of ACD and know that chances to remain self-reliance increase compared to MCB. | Fixed (written) arrangements regarding the management of adjustable compression devices as preferred compression therapy completely executed by home care organizations are available.  GPs motivation to make use of arrangements, decreased stress-levels and increased confidence that ACD are reimbursed without problems for the patient. | Adjustable compression devices are prescribed which increases the possibility to maintain self-reliant |  |
| **Inhibiting** |  |  |  |
| **DVT and CVD** |  |  |  |
| Lack of knowledge: Most GPs have insufficient knowledge regarding the different types of initial compression therapy (L). The national guideline recommends using MCB | Commitment to guideline recommendations | MCB is prescribed, patients depend on home care or outpatient clinic for assistance |  |
| Patient characteristics: Patients who have insufficient strength and/or cognitive abilities to apply and remove the initial compression therapy self-reliantly | Lack of ability | Patients depend on home care or outpatient clinic for assistance |  |
| **CVD** |  |  |  |
| Reimbursement constraints and perceived lack of evidence regarding ACD by dermatologists and internists (L + NH) and GPs (L) | Reticent attitude regarding the use of ACD | Omission of ACD |  |

Abbreviations L: Limburg, NH-A: North-Holland location A, GP: general practitioner, DVT: deep venous thrombosis, CVD: chronic venous disease, ACD: adjustable compression devices, TCH: temporary compression hosiery, MCB: multilayer compression bandages

**Selection of ECS type and class**

**Table 4:** **Information and patient transfer**

| **Context (+)** | **Mechanism (=)** | **Proximal outcome** | **Distal outcome** |
| --- | --- | --- | --- |
| **Enhancing** |  |  | Delivered ECS  **DVT**  *Custom-made*  L: 39%, NH: 33%  *Ready-made*  L: 61%, NH: 67%  *Class 2*  L: 30%, NH: 65%  *Class 3*  L: 68%, NH: 35%  *Other*  L: 2%, NH: 0%  **CVD**  *Custom-made*  L: 24%, NH: 25%  *Ready-made*  L: 76%, NH: 75%  *Class 2*  L: 77%, NH: 87%  *Class 3*  L: 23%, NH: 13% |
| **DVT** |  |  |  |
| Knowledge: Most internists and specialized GPs have sufficient knowledge to determine the indication and ECS class based on evidence (L+NH) | Willingness and commitment | Complete referrals including the ECS indication and class based on evidence |  |
| Lack of knowledge: Some internists have insufficient knowledge to determine the ECS class based on evidence. | An evidence-based preprinted recipe for class 3 ECS is available at the workplace.  Increased confidence and self-efficacy in prescribing the ECS | Complete referrals including the ECS indication and class based on evidence |  |
| **CVD** |  |  |  |
| Knowledge: Dermatologists (L) and some GPs (L+NH) have sufficient knowledge to determine the indication and ECS class based on evidence (L+NH) | Willingness and commitment | Complete referrals including the ECS indication and class based on evidence |  |
| **DVT + CVD** |  |  |  |
| Complete referrals: ECS referrals are complete and contain patient information, ECS indication, and ECS class | Increased efficacy, alignment and knowledge regarding each other’s needs and wishes  Medical stocking supplier’s confidence and commitment to the treating physician’s approach and instructions  Willingness and motivation to collaborate and improve interdisciplinary processes | Increased ECS deliveries as intended by the treating physician |  |
| Understanding of responsibilities, roles, and mutual expectations among the treating physician and the medical stocking supplier |  |  |  |
| Accessible interdisciplinary consultation |  |  |  |
| **Inhibiting** |  |  |  |
| **DVT** |  |  |  |
| Lack of knowledge: dermatologists (NH) lack evidence-based knowledge regarding how to determine the indicated ECS class for DVT. The local protocol does provide recommendations that are not evidence-based | Commitment to the local protocol | ECS class included in the referral is not evidence-based |  |
| **DVT + CVD** |  |  |  |
| Lack of knowledge/exposure: most GPs have insufficient knowledge to determine the indicated ECS class based on evidence. The national guideline provides evidence-based recommendations | Evidence regarding the ECS class is difficult to find in the national guideline  Lack of time to rigorously search the guideline and lack of self-confidence to commit to the national guideline | Incomplete referrals lacking the ECS indication and/or class |  |
| Incomplete referrals and financial constraints: if ECS referrals are incomplete and lack patient information, ECS indication, and/or ECS class the medical stocking supplier selects the indicated ECS. Medical stocking suppliers generate income based on a fixed cluster fee per patient, which is a set amount and is perceived to be insufficient  Lack of understanding of responsibilities and mutual expectations among treating physicians and medical stocking suppliers | Reticent attitude to implement expensive ECS types, increased risk of selection of ECS based on financial incentives  Lack of willingness to invest time and motivation to collaborate and improve interdisciplinary processes | Decreased ECS deliveries as intended by evidence  Lack of coordination of information transfer |  |
|  |  |  |  |

Abbreviations L: Limburg, NH: North-Holland, GP: general practitioner, DVT: deep venous thrombosis, CVD: chronic venous disease, ECS: elastic compression stockings

**Patient-based selection of assistive devices**

**Table 5:** **Selection and training of assistive devices**

| **Context (+)** | **Mechanism (=)** | **Proximal outcome** | **Intermediate outcome** | **Distal outcome** |
| --- | --- | --- | --- | --- |
| **Enhancing** |  |  |  | **DVT**  *Self-reliant without AD*  L: 23%, NH: 50%  *Self-reliant with AD without training*  L: 56%, NH: 35%  *Self-reliant with AD after training*  L: 11%, NH: 8%  *Long term home care for ECS*  L: 10%, NH: 7%  **CVD**  *Self-reliant without AD*  L: 25%, NH: 33%  *Self-reliant with AD without training*  L: 45%, NH: 53%  *Self-reliant with AD after training*  L: 14%, NH: 7%  *Long term home care for ECS*  L: 16%, NH: 7% |
| **DVT and CVD** |  |  |  |  |
| Patient characteristics: younger patient’s generally possess more strength and cognitive abilities | Motivation and ability to use their abilities | - | Increased number of self-reliant patients |  |
| Informal caregiver: availability of an informal caregiver which can support and train the patient with the use of an AD | Active involvement of the caregiver in the process.  Patients are feeling in control and safe since their caregiver is informed to support them. | Less referrals to home care, less/no additional training needed | Increased implementation rate AD, increased number of self-reliant patients |  |
| Patient’s knowledge and perspectives: appropriately informed patients who are aware of their desires and aims, and aspire to maintain self-reliance | Active patient involvement in the process.  Patients are feeling in control of the situation and are more confident in directing care. | Patient trains most suitable AD | Increased implementation rate AD, increased number of self-reliant patients |  |
| Time constraints: occupational therapists have sufficient time to select and train AD | Patient-based adjustment of time | Training duration based on patient’s needs | Increased implementation rate AD, increased number of self-reliant patients |  |
| Trained staff*: occupational therapists are sufficiently trained to select and train AD based on patient’s characteristics; desires and aims. | Patient-based selection of AD | Patient trains most suitable AD | Increased implementation rate AD, increased number of self-reliant patients |  |
| Accessible interdisciplinary consultation*: direct peer consultation among medical stocking suppliers and occupational therapists | Increased efficacy, alignment and knowledge regarding each other’s needs  Willingness and ability to collaborate and improve interdisciplinary processes  Improved continuity of care | The occupational therapist is better informed of the patient’s situation | Increased implementation rate AD, increased number of self-reliant patients |  |
| **Inhibiting** |  |  |  |  |
| **DVT and CVD** |  |  |  |  |
| Time constraints:  1. Medical stocking suppliers (L+NH) and 2. Home care nurses (NH+L) experience high time pressure and workload demands | Prioritizing tasks, reticent attitude to invest time | 1. Short explanation of AD/omission of implementation AD  2. Decreased training time | 1. Increased number of referrals to home care and occupational therapists  2. Decreased implementation rate AD |  |
| Financial constraints: Training consults provided by the medical stocking supplier are financially included in the cluster fee, which is not extended if additional time is invested | Reticent attitude to invest time for training | Increased referrals to home care and occupational therapist | - |  |
| Patient characteristics: elderly and obese patients are less likely to apply and remove the ECS self-reliantly | Lack of strength and physical abilities | - | Decreased implementation rate AD |  |
| Lack of patient’s knowledge regarding different assistive devices and their aims and desires regarding these assistive devices | Patients not actively involved in the decision-making process or patients are feeling overwhelmed by the variety of choices which makes them feel loosing control | Higher risk to select an AD less suitable for the patient | Decreased implementation rate AD |  |
| Lack of understanding of responsibilities and mutual expectations among involved professionals | Lack of willingness and motivation to collaborate and improve interdisciplinary processes | Lack of coordination of patient transfer | Decreased implementation rate AD |  |
| Lack of trained staff*: home care nurses are not sufficiently trained to select and train AD based on patient’s characteristics; desires and aims (L) | Selection of AD based on personal experience | Higher risk to select an AD less suitable for the patient | Decreased implementation rate AD, decreased number of self-reliant patients |  |
| Lack of accessible interdisciplinary consultation*: lack of communication among medical stocking suppliers and home care nurses to discuss options to maintain patient’s self-reliance. It is perceived to be difficult and time-consuming to contact each other. | Lack of willingness and motivation to collaborate | Home care nurses are suboptimally informed/prepared for the patient’s situation | Decreased implementation rate AD |  |
| Patient characteristics*: occupational therapists and home care nurses perceive it to be more difficult to encourage patients to be self-reliant when they were already used to home care. | Patients feel anxious that the use of an assistive device indicates they will lose home care contacts and are not motivated for training | Decreased patients selected for training | Decreased implementation rate AD |  |

* Context factors related to patients who require additional training

Abbreviations L: Limburg, NH: North-Holland, AD: assistive device, DVT: deep venous thrombosis, CVD: chronic venous disease

**Individualized treatment duration for DVT patients**

**Table 6:** **Individualized duration of ECS therapy for DVT patients**

| **Context (+)** | **Mechanism (=)** | **Proximal outcome** | **Distal outcome** |
| --- | --- | --- | --- |
| **Enhancing** |  |  | *Standardized treatment duration*  L: 17%, NH: 100%  *Individualized treatment duration*  L: 83%, NH: 0%  *Individualized ECS treatment duration*  L: 100%, NH: 0% |
| Knowledge: Most internists have sufficient knowledge regarding the individualization of treatment duration for DVT-patients, value the added value for the patient, and how to determine Villalta scores (L) | Confidence and motivation to individualize treatment duration | Individualized ECS treatment duration |  |
| Lack of knowledge: Some internists lack knowledge regarding the possibility to individualize treatment duration (L). They are aware of the local protocol providing unambiguous recommendations to individualize the ECS treatment duration | Increased self-confidence and commitment to use the local protocol | Individualized ECS treatment duration |  |
| Perspectives: internists feel responsible for leg assessments and risk assessment for the post-thrombotic syndrome (L) | Internists motivation and willingness to invest time | Individualized ECS treatment duration |  |
| Lack of knowledge: Internists (NH) and GPs (L) lack knowledge, experience, and training in estimating Villalta-scores since they rarely assess legs. A tool is available to stepwise guide health care professionals on how to determine a Villalta score | Increased self-confidence and self-efficacy to determine Villalta-scores. | Individualized ECS treatment duration |  |
| **Inhibiting** |  |  |  |
| Lack of knowledge: GPs lack knowledge regarding the possibility to individualize treatment duration (L+NH). The national GPs guideline recommends a standardized treatment duration and does not provide support on how to individualize ECS treatment duration  Internists and dermatologists are aware of the possibility to individualize treatment duration, however, they have insufficient knowledge of the content of this approach (NH). The local protocol recommends a standardized treatment duration and does not provide support on how to individualize ECS treatment duration | Rely back on acquired (lack of) knowledge | Standardized treatment duration without formal leg assessments |  |
| Lack of knowledge: Internists (NH) and GPs (L) lack knowledge, experience, and training in estimating Villalta-scores since they rarely assess legs | Reticent attitude and lack of self-confidence to determine Villalta scores | Standardized ECS treatment duration without formal leg assessments |  |
| Perspectives: internists experience leg assessments and risk assessment for the post-thrombotic syndrome as a dermatological problem and do not feel responsible for this follow-up (NH) | Lack of motivation and willingness to invest time for follow-up | Standardized ECS treatment duration without formal leg assessments |  |
| Time constraints: dermatologists’ and internists’ outpatient clinics are already fully booked, they experience a lack of time to perform more follow-up consults (NH) | Lack of motivation and willingness to invest time for follow-up | Standardized ECS treatment duration without formal leg assessments |  |
| Lack of interdisciplinary communication: among the indicated ECS treatment duration among internists (L, NH-B) or dermatologists (NH-A), and medical stocking suppliers (L+NH). | Medical stocking suppliers send patients annual calls for follow-up regardless of the advised treatment duration.  Patient’s interpretation of the automated call as an indication to restart ECS therapy | Some patients resume ECS therapy regardless of the advised treatment duration |  |

Abbreviations L: Limburg, NH: North-Holland, NH-B: North-Holland location B, GP: general practitioner, DVT: deep venous thrombosis, ECS: elastic compression stockings

**Provision of follow-up for CVD patients**

**Table 7:** **Performing follow-up for CVD patients**

| **Context (+)** | **Mechanism (=)** | **Distal outcome** |
| --- | --- | --- |
| **Enhancing** |  | *FU only if problems exist*  *GP*  L: 42%, NH: 62%  *Dermatologist*  L/NH: 0%  *FU by default*  *GP*  L: 58%, NH: 38%  *Dermatologist*  L/NH: 100% |
| Perspective: Dermatologists and approximately half of GPs value the need for a follow-up appointment to check adherence to therapy and provide additional information (L+NH) | Motivation and willingness to invest time |  |
| Patient characteristics: In general, patients visiting the dermatologists have more severe, or complicated disease (L+NH) | Motivation and willingness to invest time |  |
| **Inhibiting** |  |  |
| Time constraints: Most GPs experience a high workload, and outpatient clinic appointments are already fully booked (L+NH) | Prioritizing tasks, reticent attitude to perform follow-up |  |
| Understanding of responsibilities, roles, and mutual: Half of GPs do not value the need for a follow-up appointment. As they experience that, for most patients, different healthcare professionals (i.e. edema therapists or home care nurses) are involved in ECS therapy. These professionals monitor the process and contact the GP if problems exist. However, these professionals indicate patient problems that they cannot solve without interference of the GP | Lack of GPs willingness and involvement in follow-up |  |

Abbreviations L: Limburg, NH: North-Holland, GP: general practitioner, CVD: chronic venous disease
